# Supplementary material for: Hot spot prediction in protein-protein interactions by an ensemble system
Source: BMC Syst Biol. 2018 Dec 31;12(Suppl 9):132. doi: 10.1186/s12918-018-0665-8 (PMC6311905; doi:10.1186/s12918-018-0665-8)
Supplement: Supplementary file 2 — All relevant MATLAB code. A simple Matlab implement of our predictor. (ZIP 19,742 kb) [file 12918_2018_665_MOESM2_ESM.zip › Additional file 2/Read ASA/ASA website/dbMPIKT/4EKCA.html]

 NetSurfP - Protein Surface Accessibility and Secondary Structure Predictions

  

|  |  |  |
| --- | --- | --- |
|  |  | NetSurfP - Protein Surface Accessibility and Secondary Structure Predictions  Technical University of Denmark |

  


---

```
# For publication of results, please cite:
# A generic method for assignment of reliability scores applied to solvent accessibility predictions.
# Bent Petersen, Thomas Nordahl Petersen, Pernille Andersen, Morten Nielsen and Claus Lundegaard
# BMC Structural Biology 2009, 9:51 doi:10.1186/1472-6807-9-51
#
# Column 1: Class assignment - B for buried or E for Exposed - Threshold: 25% exposure, but not based on RSA
# Column 2: Amino acid
# Column 3: Sequence name
# Column 4: Amino acid number
# Column 5: Relative Surface Accessibility - RSA
# Column 6: Absolute Surface Accessibility
# Column 7: Z-fit score for RSA prediction
# Column 8: Probability for Alpha-Helix
# Column 9: Probability for Beta-strand
# Column 10: Probability for Coil
E G  4EKCA                  1    0.844  66.399  -0.572   0.003   0.003   0.994
E A  4EKCA                  2    0.705  77.669  -0.026   0.502   0.002   0.495
E M  4EKCA                  3    0.645 129.065  -0.288   0.858   0.002   0.139
E G  4EKCA                  4    0.522  41.042   0.577   0.858   0.002   0.139
E S  4EKCA                  5    0.435  50.935   0.478   0.858   0.002   0.139
B A  4EKCA                  6    0.285  31.352  -0.284   0.858   0.002   0.139
E R  4EKCA                  7    0.480 109.897   1.347   0.923   0.002   0.076
E R  4EKCA                  8    0.598 136.965   1.520   0.923   0.002   0.076
E I  4EKCA                  9    0.294  54.353   0.897   0.923   0.002   0.076
B N  4EKCA                 10    0.126  18.476  -0.129   0.782   0.003   0.216
E D  4EKCA                 11    0.553  79.702   1.575   0.858   0.002   0.139
E E  4EKCA                 12    0.549  95.893   1.864   0.923   0.002   0.076
B I  4EKCA                 13    0.047   8.787   0.637   0.923   0.002   0.076
B E  4EKCA                 14    0.205  35.883   0.641   0.923   0.002   0.076
E R  4EKCA                 15    0.574 131.469   1.661   0.970   0.001   0.030
E Q  4EKCA                 16    0.370  66.046   1.228   0.970   0.001   0.030
B L  4EKCA                 17    0.047   8.661   0.897   0.970   0.001   0.030
E R  4EKCA                 18    0.518 118.599   1.619   0.970   0.001   0.030
E R  4EKCA                 19    0.587 134.492   1.579   0.923   0.002   0.076
E D  4EKCA                 20    0.309  44.498   0.253   0.923   0.002   0.076
E K  4EKCA                 21    0.330  67.860   0.262   0.923   0.002   0.076
E R  4EKCA                 22    0.602 137.904   0.779   0.938   0.007   0.055
E D  4EKCA                 23    0.564  81.287   0.491   0.879   0.010   0.111
E A  4EKCA                 24    0.287  31.660  -0.412   0.717   0.014   0.269
E R  4EKCA                 25    0.492 112.668   0.318   0.717   0.014   0.269
E R  4EKCA                 26    0.496 113.607   0.568   0.522   0.016   0.462
E E  4EKCA                 27    0.396  69.216   1.510   0.386   0.097   0.517
B L  4EKCA                 28    0.066  12.030   0.790   0.148   0.418   0.435
B K  4EKCA                 29    0.208  42.786   1.566   0.087   0.683   0.230
B L  4EKCA                 30    0.023   4.138   0.993   0.018   0.846   0.136
B L  4EKCA                 31    0.033   6.134   0.908   0.011   0.918   0.071
B L  4EKCA                 32    0.018   3.296   1.172   0.011   0.918   0.071
B L  4EKCA                 33    0.029   5.255   0.508   0.021   0.756   0.223
B G  4EKCA                 34    0.043   3.353  -0.715   0.005   0.336   0.660
B T  4EKCA                 35    0.120  16.644  -0.294   0.004   0.085   0.910
E G  4EKCA                 36    0.282  22.201  -1.034   0.018   0.019   0.964
E E  4EKCA                 37    0.425  74.282  -0.854   0.058   0.017   0.925
B S  4EKCA                 38    0.074   8.731  -1.539   0.115   0.016   0.868
B G  4EKCA                 39    0.075   5.910  -1.421   0.176   0.004   0.820
B K  4EKCA                 40    0.153  31.534  -0.667   0.694   0.003   0.303
B S  4EKCA                 41    0.150  17.627  -0.419   0.782   0.003   0.216
B T  4EKCA                 42    0.182  25.202   0.356   0.923   0.002   0.076
B F  4EKCA                 43    0.049   9.915   0.111   0.970   0.001   0.030
B I  4EKCA                 44    0.058  10.767   0.731   0.970   0.001   0.030
E K  4EKCA                 45    0.302  62.183   0.805   0.975   0.003   0.022
B Q  4EKCA                 46    0.140  24.968  -0.051   0.970   0.001   0.030
B M  4EKCA                 47    0.034   6.883   0.459   0.938   0.007   0.055
E R  4EKCA                 48    0.265  60.708   1.569   0.938   0.007   0.055
E I  4EKCA                 49    0.231  42.698   0.475   0.938   0.007   0.055
B I  4EKCA                 50    0.132  24.346   0.586   0.879   0.010   0.111
B H  4EKCA                 51    0.185  33.724   0.463   0.502   0.002   0.495
E G  4EKCA                 52    0.437  34.392   0.055   0.109   0.005   0.886
E S  4EKCA                 53    0.699  81.888  -0.399   0.016   0.005   0.979
E G  4EKCA                 54    0.628  49.447  -1.321   0.003   0.003   0.994
B Y  4EKCA                 55    0.191  40.731  -0.490   0.016   0.005   0.979
E S  4EKCA                 56    0.422  49.482   0.399   0.016   0.005   0.979
E D  4EKCA                 57    0.587  84.659   0.802   0.923   0.002   0.076
E E  4EKCA                 58    0.736 128.649   1.047   0.923   0.002   0.076
B D  4EKCA                 59    0.178  25.679  -0.330   0.923   0.002   0.076
B K  4EKCA                 60    0.124  25.425   0.460   0.923   0.002   0.076
E R  4EKCA                 61    0.446 102.065   0.837   0.923   0.002   0.076
E G  4EKCA                 62    0.529  41.617   0.574   0.858   0.002   0.139
B F  4EKCA                 63    0.080  16.136   0.799   0.858   0.002   0.139
B T  4EKCA                 64    0.138  19.113   0.724   0.923   0.002   0.076
E K  4EKCA                 65    0.392  80.552   0.362   0.970   0.001   0.030
B L  4EKCA                 66    0.144  26.311   1.167   0.970   0.001   0.030
B V  4EKCA                 67    0.016   2.413   1.523   0.975   0.003   0.022
B Y  4EKCA                 68    0.122  26.050   0.414   0.970   0.001   0.030
E Q  4EKCA                 69    0.361  64.439   1.155   0.970   0.001   0.030
B N  4EKCA                 70    0.058   8.550   0.393   0.970   0.001   0.030
B I  4EKCA                 71    0.019   3.534   1.056   0.970   0.001   0.030
B F  4EKCA                 72    0.115  23.040   0.064   0.970   0.001   0.030
E T  4EKCA                 73    0.317  43.968   0.849   0.970   0.001   0.030
B A  4EKCA                 74    0.035   3.868  -1.508   0.970   0.001   0.030
B M  4EKCA                 75    0.023   4.702   0.034   0.970   0.001   0.030
E Q  4EKCA                 76    0.272  48.651   0.250   0.970   0.001   0.030
E A  4EKCA                 77    0.247  27.230   0.441   0.970   0.001   0.030
B M  4EKCA                 78    0.024   4.742   0.860   0.970   0.001   0.030
B I  4EKCA                 79    0.059  10.897   0.016   0.970   0.001   0.030
E R  4EKCA                 80    0.385  88.188   1.109   0.988   0.000   0.012
B A  4EKCA                 81    0.097  10.711  -1.131   0.970   0.001   0.030
B M  4EKCA                 82    0.080  15.948  -1.208   0.970   0.001   0.030
E D  4EKCA                 83    0.569  82.036  -0.021   0.923   0.002   0.076
E T  4EKCA                 84    0.558  77.450   0.165   0.782   0.003   0.216
B L  4EKCA                 85    0.229  41.912  -0.374   0.406   0.004   0.590
E K  4EKCA                 86    0.720 148.125  -0.275   0.053   0.005   0.942
B I  4EKCA                 87    0.168  31.099  -0.151   0.018   0.019   0.964
E P  4EKCA                 88    0.645  91.596   0.592   0.018   0.047   0.935
B Y  4EKCA                 89    0.174  37.205  -0.577   0.018   0.047   0.935
E K  4EKCA                 90    0.718 147.734   0.031   0.018   0.019   0.964
E Y  4EKCA                 91    0.486 103.794  -0.748   0.115   0.016   0.868
E E  4EKCA                 92    0.637 111.266  -0.695   0.430   0.016   0.555
E H  4EKCA                 93    0.579 105.284  -1.103   0.406   0.004   0.590
E N  4EKCA                 94    0.375  54.841  -0.398   0.694   0.003   0.303
E K  4EKCA                 95    0.547 112.518   0.116   0.858   0.002   0.139
E A  4EKCA                 96    0.492  54.218   0.267   0.879   0.010   0.111
B H  4EKCA                 97    0.220  40.000   0.456   0.879   0.010   0.111
B A  4EKCA                 98    0.127  14.017  -1.256   0.879   0.010   0.111
E Q  4EKCA                 99    0.537  95.819   0.855   0.911   0.033   0.057
B L  4EKCA                100    0.166  30.376   1.083   0.911   0.033   0.057
B V  4EKCA                101    0.115  17.614  -0.821   0.870   0.077   0.053
E R  4EKCA                102    0.361  82.577   0.059   0.779   0.100   0.120
E E  4EKCA                103    0.407  71.050  -0.835   0.605   0.105   0.290
B V  4EKCA                104    0.186  28.604   0.048   0.268   0.043   0.689
E D  4EKCA                105    0.538  77.583  -1.270   0.113   0.043   0.844
E V  4EKCA                106    0.401  61.695  -2.059   0.115   0.016   0.868
E E  4EKCA                107    0.612 106.916  -1.873   0.115   0.016   0.868
E K  4EKCA                108    0.644 132.491  -1.295   0.115   0.016   0.868
E V  4EKCA                109    0.391  60.035  -1.287   0.115   0.016   0.868
E S  4EKCA                110    0.420  49.283  -1.016   0.058   0.017   0.925
E A  4EKCA                111    0.487  53.667  -1.119   0.115   0.016   0.868
B F  4EKCA                112    0.086  17.340  -0.695   0.115   0.016   0.868
B D  4EKCA                113    0.189  27.206  -0.539   0.406   0.004   0.590
E V  4EKCA                114    0.509  78.233   0.109   0.858   0.002   0.139
E P  4EKCA                115    0.490  69.602   0.624   0.923   0.002   0.076
B D  4EKCA                116    0.039   5.562  -0.037   0.970   0.001   0.030
B Y  4EKCA                117    0.081  17.331   0.180   0.970   0.001   0.030
E A  4EKCA                118    0.560  61.734   1.438   0.970   0.001   0.030
B A  4EKCA                119    0.128  14.095   0.964   0.970   0.001   0.030
B I  4EKCA                120    0.013   2.497   1.306   0.970   0.001   0.030
E K  4EKCA                121    0.347  71.481   1.253   0.923   0.002   0.076
E S  4EKCA                122    0.438  51.322   1.377   0.879   0.010   0.111
B L  4EKCA                123    0.060  11.059   0.813   0.782   0.003   0.216
B W  4EKCA                124    0.101  24.411  -0.205   0.502   0.002   0.495
E N  4EKCA                125    0.609  89.202  -0.477   0.176   0.004   0.820
B D  4EKCA                126    0.225  32.408  -1.144   0.053   0.005   0.942
E P  4EKCA                127    0.659  93.470  -0.091   0.600   0.003   0.397
B G  4EKCA                128    0.243  19.108  -1.221   0.694   0.003   0.303
B I  4EKCA                129    0.044   8.214  -0.039   0.879   0.010   0.111
E Q  4EKCA                130    0.271  48.418   1.130   0.938   0.007   0.055
E E  4EKCA                131    0.396  69.164   1.375   0.938   0.007   0.055
B C  4EKCA                132    0.037   5.167  -0.414   0.938   0.007   0.055
B Y  4EKCA                133    0.122  26.007   0.252   0.879   0.010   0.111
E D  4EKCA                134    0.567  81.748   0.364   0.802   0.014   0.185
E R  4EKCA                135    0.429  98.218  -0.002   0.430   0.016   0.555
E R  4EKCA                136    0.417  95.493  -0.842   0.115   0.016   0.868
E R  4EKCA                137    0.496 113.492  -0.518   0.053   0.043   0.903
E E  4EKCA                138    0.511  89.237  -0.215   0.052   0.084   0.864
B Y  4EKCA                139    0.085  18.079  -0.001   0.019   0.141   0.840
E Q  4EKCA                140    0.429  76.530   0.530   0.020   0.205   0.775
B L  4EKCA                141    0.079  14.538   0.244   0.019   0.141   0.840
E S  4EKCA                142    0.333  39.051  -1.029   0.018   0.047   0.935
E D  4EKCA                143    0.346  49.902  -1.470   0.115   0.016   0.868
B S  4EKCA                144    0.162  18.998  -1.203   0.246   0.004   0.750
B T  4EKCA                145    0.077  10.624  -0.577   0.600   0.003   0.397
E K  4EKCA                146    0.412  84.646   0.129   0.782   0.003   0.216
B Y  4EKCA                147    0.191  40.774   0.201   0.802   0.014   0.185
B Y  4EKCA                148    0.071  15.130   0.080   0.802   0.014   0.185
B L  4EKCA                149    0.085  15.527  -0.114   0.858   0.002   0.139
E N  4EKCA                150    0.482  70.536   0.670   0.858   0.002   0.139
B D  4EKCA                151    0.172  24.800   0.441   0.858   0.002   0.139
B L  4EKCA                152    0.033   6.134  -0.382   0.923   0.002   0.076
E D  4EKCA                153    0.312  44.988   0.073   0.923   0.002   0.076
E R  4EKCA                154    0.369  84.570   1.031   0.923   0.002   0.076
B V  4EKCA                155    0.058   8.945  -0.115   0.879   0.010   0.111
B A  4EKCA                156    0.186  20.508   0.274   0.717   0.014   0.269
E D  4EKCA                157    0.449  64.686   0.485   0.321   0.003   0.675
E P  4EKCA                158    0.576  81.777  -0.394   0.109   0.005   0.886
E S  4EKCA                159    0.668  78.243  -0.966   0.058   0.017   0.925
B Y  4EKCA                160    0.084  17.865   0.076   0.018   0.088   0.893
E L  4EKCA                161    0.367  67.216   0.919   0.020   0.205   0.775
B P  4EKCA                162    0.157  22.264  -0.167   0.020   0.205   0.775
E T  4EKCA                163    0.256  35.563   0.655   0.052   0.084   0.864
E Q  4EKCA                164    0.551  98.444  -0.877   0.113   0.043   0.844
E Q  4EKCA                165    0.697 124.395  -0.854   0.058   0.017   0.925
E D  4EKCA                166    0.292  42.106  -0.796   0.053   0.043   0.903
E V  4EKCA                167    0.419  64.446  -0.739   0.052   0.084   0.864
B L  4EKCA                168    0.264  48.283  -0.702   0.064   0.216   0.721
E R  4EKCA                169    0.443 101.539  -1.047   0.022   0.359   0.619
B V  4EKCA                170    0.144  22.117  -0.395   0.022   0.552   0.426
E C  4EKCA                171    0.445  62.492  -0.479   0.021   0.451   0.528
B V  4EKCA                172    0.126  19.351  -0.217   0.005   0.262   0.733
B P  4EKCA                173    0.241  34.127  -0.269   0.004   0.138   0.858
B T  4EKCA                174    0.095  13.176  -1.008   0.004   0.420   0.576
B T  4EKCA                175    0.170  23.579   0.239   0.004   0.616   0.381
B G  4EKCA                176    0.092   7.272  -1.387   0.004   0.616   0.381
B I  4EKCA                177    0.072  13.302  -0.084   0.002   0.816   0.182
B I  4EKCA                178    0.141  26.159  -0.083   0.001   0.900   0.099
B E  4EKCA                179    0.207  36.198  -0.283   0.001   0.900   0.099
B Y  4EKCA                180    0.217  46.287   0.297   0.001   0.959   0.040
E P  4EKCA                181    0.366  51.907   0.871   0.001   0.959   0.040
B F  4EKCA                182    0.046   9.292   0.415   0.001   0.900   0.099
E D  4EKCA                183    0.463  66.704   0.484   0.001   0.900   0.099
B L  4EKCA                184    0.148  27.154  -0.512   0.004   0.514   0.481
E Q  4EKCA                185    0.669 119.412  -0.306   0.005   0.045   0.951
E S  4EKCA                186    0.700  82.040  -0.572   0.005   0.015   0.979
B V  4EKCA                187    0.131  20.181   1.048   0.002   0.816   0.182
E I  4EKCA                188    0.362  66.896   0.783   0.001   0.900   0.099
B F  4EKCA                189    0.052  10.497   0.725   0.001   0.900   0.099
B R  4EKCA                190    0.122  27.961  -0.127   0.018   0.846   0.136
B M  4EKCA                191    0.029   5.703  -0.836   0.021   0.756   0.223
B V  4EKCA                192    0.162  24.930  -0.015   0.003   0.718   0.279
B D  4EKCA                193    0.061   8.790  -2.873   0.004   0.420   0.576
B V  4EKCA                194    0.044   6.747  -0.927   0.020   0.205   0.775
B G  4EKCA                195    0.127   9.987  -2.300   0.018   0.088   0.893
B G  4EKCA                196    0.211  16.582  -2.310   0.018   0.019   0.964
E Q  4EKCA                197    0.346  61.706  -0.453   0.018   0.019   0.964
E R  4EKCA                198    0.495 113.263  -0.822   0.053   0.043   0.903
E S  4EKCA                199    0.493  57.768  -1.721   0.053   0.043   0.903
B E  4EKCA                200    0.192  33.490  -1.242   0.113   0.043   0.844
B R  4EKCA                201    0.254  58.166  -0.489   0.184   0.043   0.773
E R  4EKCA                202    0.440 100.760  -0.989   0.278   0.093   0.628
B K  4EKCA                203    0.258  53.029  -0.776   0.386   0.097   0.517
B W  4EKCA                204    0.043  10.341  -0.407   0.502   0.102   0.396
E I  4EKCA                205    0.285  52.780   0.645   0.605   0.105   0.290
E H  4EKCA                206    0.415  75.525   0.048   0.502   0.102   0.396
B C  4EKCA                207    0.089  12.453  -0.397   0.502   0.102   0.396
B F  4EKCA                208    0.054  10.798  -0.204   0.191   0.086   0.723
E E  4EKCA                209    0.554  96.766  -0.970   0.052   0.084   0.864
E N  4EKCA                210    0.442  64.738  -0.890   0.018   0.047   0.935
B V  4EKCA                211    0.104  15.939  -0.115   0.005   0.045   0.951
B T  4EKCA                212    0.205  28.503  -0.335   0.004   0.197   0.799
B S  4EKCA                213    0.023   2.707   0.506   0.002   0.816   0.182
B I  4EKCA                214    0.015   2.849   1.409   0.001   0.959   0.040
B M  4EKCA                215    0.013   2.561   1.695   0.001   0.959   0.040
B F  4EKCA                216    0.021   4.235   1.391   0.001   0.959   0.040
B L  4EKCA                217    0.012   2.216   1.692   0.001   0.900   0.099
B V  4EKCA                218    0.024   3.643   0.775   0.003   0.718   0.279
B A  4EKCA                219    0.057   6.248  -1.806   0.004   0.420   0.576
B L  4EKCA                220    0.043   7.928  -1.424   0.005   0.045   0.951
B S  4EKCA                221    0.102  11.908  -1.179   0.018   0.019   0.964
B E  4EKCA                222    0.243  42.435  -0.809   0.058   0.017   0.925
E Y  4EKCA                223    0.305  65.178  -0.817   0.058   0.017   0.925
E D  4EKCA                224    0.345  49.715  -1.648   0.052   0.084   0.864
B Q  4EKCA                225    0.274  48.990  -0.041   0.069   0.386   0.545
B V  4EKCA                226    0.227  34.859  -0.955   0.074   0.484   0.442
B L  4EKCA                227    0.230  42.131  -0.364   0.159   0.516   0.325
E V  4EKCA                228    0.390  60.004  -0.620   0.148   0.418   0.435
E E  4EKCA                229    0.461  80.554  -1.366   0.231   0.330   0.439
E S  4EKCA                230    0.315  36.941  -0.709   0.199   0.152   0.649
E D  4EKCA                231    0.550  79.298  -0.711   0.191   0.086   0.723
E N  4EKCA                232    0.545  79.817  -1.038   0.268   0.043   0.689
E E  4EKCA                233    0.355  62.036   0.578   0.268   0.043   0.689
B N  4EKCA                234    0.267  39.045  -0.703   0.184   0.043   0.773
B R  4EKCA                235    0.173  39.640   1.425   0.622   0.015   0.363
B M  4EKCA                236    0.040   7.984  -0.023   0.938   0.007   0.055
E E  4EKCA                237    0.437  76.379   1.263   0.970   0.001   0.030
E E  4EKCA                238    0.282  49.213   1.097   0.970   0.001   0.030
B S  4EKCA                239    0.016   1.910   0.781   0.970   0.001   0.030
B K  4EKCA                240    0.155  31.842   1.140   0.970   0.001   0.030
E A  4EKCA                241    0.562  61.965   1.566   0.970   0.001   0.030
B L  4EKCA                242    0.117  21.459   1.188   0.988   0.000   0.012
B F  4EKCA                243    0.019   3.894   0.983   0.988   0.000   0.012
E R  4EKCA                244    0.372  85.234   1.859   0.970   0.001   0.030
E T  4EKCA                245    0.438  60.737   1.775   0.970   0.001   0.030
B I  4EKCA                246    0.041   7.622   0.466   0.923   0.002   0.076
B I  4EKCA                247    0.072  13.302   0.490   0.782   0.003   0.216
E T  4EKCA                248    0.637  88.394   0.567   0.406   0.004   0.590
E Y  4EKCA                249    0.306  65.435   0.344   0.109   0.005   0.886
E P  4EKCA                250    0.693  98.337   0.463   0.109   0.005   0.886
E W  4EKCA                251    0.320  77.032   0.354   0.181   0.016   0.803
B F  4EKCA                252    0.050  10.015  -0.471   0.257   0.016   0.727
E Q  4EKCA                253    0.424  75.762   1.163   0.115   0.016   0.868
E N  4EKCA                254    0.714 104.544   1.022   0.018   0.019   0.964
B S  4EKCA                255    0.034   3.938   1.201   0.018   0.088   0.893
B S  4EKCA                256    0.070   8.263   0.671   0.021   0.451   0.528
B V  4EKCA                257    0.023   3.474   1.253   0.011   0.918   0.071
B I  4EKCA                258    0.018   3.349   1.647   0.006   0.962   0.032
B L  4EKCA                259    0.017   3.094   1.592   0.011   0.918   0.071
B F  4EKCA                260    0.025   4.937   1.007   0.011   0.918   0.071
B L  4EKCA                261    0.018   3.241   0.975   0.021   0.756   0.223
B N  4EKCA                262    0.084  12.371   0.446   0.022   0.359   0.619
B K  4EKCA                263    0.166  34.126   0.821   0.191   0.086   0.723
E K  4EKCA                264    0.234  48.072   0.863   0.354   0.048   0.598
E D  4EKCA                265    0.261  37.567  -0.411   0.522   0.016   0.462
B L  4EKCA                266    0.162  29.571   0.150   0.717   0.014   0.269
B L  4EKCA                267    0.086  15.783   0.106   0.717   0.014   0.269
E E  4EKCA                268    0.490  85.603   1.425   0.717   0.014   0.269
E E  4EKCA                269    0.649 113.310   0.601   0.717   0.014   0.269
E K  4EKCA                270    0.284  58.378   0.746   0.622   0.015   0.363
B I  4EKCA                271    0.078  14.411  -0.265   0.430   0.016   0.555
E M  4EKCA                272    0.542 108.374   0.545   0.181   0.016   0.803
E Y  4EKCA                273    0.623 133.071   0.362   0.113   0.043   0.844
B S  4EKCA                274    0.218  25.561   0.245   0.053   0.043   0.903
E H  4EKCA                275    0.554 100.791   0.686   0.018   0.047   0.935
B L  4EKCA                276    0.061  11.224  -0.393   0.056   0.142   0.802
E V  4EKCA                277    0.446  68.489   0.884   0.064   0.216   0.721
E D  4EKCA                278    0.648  93.420   0.783   0.066   0.296   0.638
B Y  4EKCA                279    0.194  41.351   0.444   0.064   0.216   0.721
B F  4EKCA                280    0.101  20.331   0.511   0.018   0.088   0.893
E P  4EKCA                281    0.484  68.736  -0.602   0.018   0.047   0.935
E E  4EKCA                282    0.658 114.970  -0.371   0.018   0.047   0.935
B Y  4EKCA                283    0.162  34.577  -0.929   0.018   0.047   0.935
E D  4EKCA                284    0.665  95.841  -0.766   0.018   0.019   0.964
E G  4EKCA                285    0.553  43.545  -1.897   0.016   0.005   0.979
E P  4EKCA                286    0.574  81.436  -1.614   0.018   0.019   0.964
E Q  4EKCA                287    0.498  88.943  -1.439   0.018   0.019   0.964
E R  4EKCA                288    0.356  81.524  -0.707   0.018   0.019   0.964
E D  4EKCA                289    0.256  36.918  -0.151   0.115   0.016   0.868
B A  4EKCA                290    0.113  12.486  -0.253   0.923   0.002   0.076
E Q  4EKCA                291    0.555  99.069   0.291   0.970   0.001   0.030
E A  4EKCA                292    0.309  34.041   0.981   0.970   0.001   0.030
B A  4EKCA                293    0.017   1.873   0.798   0.970   0.001   0.030
B R  4EKCA                294    0.119  27.297   0.633   0.970   0.001   0.030
E E  4EKCA                295    0.463  80.973   1.648   0.970   0.001   0.030
B F  4EKCA                296    0.110  22.157   1.376   0.988   0.000   0.012
B I  4EKCA                297    0.031   5.680   0.603   0.988   0.000   0.012
B L  4EKCA                298    0.185  33.947   1.142   0.988   0.000   0.012
E K  4EKCA                299    0.497 102.295   1.239   0.988   0.000   0.012
B M  4EKCA                300    0.216  43.262   1.510   0.970   0.001   0.030
B F  4EKCA                301    0.052  10.336   0.247   0.970   0.001   0.030
E V  4EKCA                302    0.370  56.915   1.252   0.923   0.002   0.076
E D  4EKCA                303    0.571  82.267   0.178   0.858   0.002   0.139
B L  4EKCA                304    0.165  30.285  -0.171   0.694   0.003   0.303
B N  4EKCA                305    0.327  47.814  -0.274   0.321   0.003   0.675
E P  4EKCA                306    0.610  86.545   0.114   0.257   0.016   0.727
E D  4EKCA                307    0.496  71.416  -0.307   0.058   0.017   0.925
E S  4EKCA                308    0.635  74.445  -0.498   0.016   0.005   0.979
E D  4EKCA                309    0.762 109.775  -0.062   0.005   0.015   0.979
E K  4EKCA                310    0.231  47.578   0.704   0.004   0.085   0.910
E I  4EKCA                311    0.356  65.879   1.441   0.004   0.616   0.381
B I  4EKCA                312    0.026   4.865   0.938   0.001   0.900   0.099
B Y  4EKCA                313    0.062  13.164   0.849   0.001   0.959   0.040
B S  4EKCA                314    0.039   4.524   0.200   0.001   0.900   0.099
B H  4EKCA                315    0.066  12.060   0.849   0.001   0.900   0.099
B F  4EKCA                316    0.072  14.370   0.089   0.018   0.846   0.136
B T  4EKCA                317    0.038   5.229  -0.093   0.021   0.756   0.223
B C  4EKCA                318    0.063   8.845  -0.069   0.021   0.756   0.223
B A  4EKCA                319    0.034   3.725  -1.058   0.022   0.552   0.426
B T  4EKCA                320    0.143  19.862   0.373   0.021   0.279   0.699
B D  4EKCA                321    0.196  28.316  -0.131   0.019   0.141   0.840
E T  4EKCA                322    0.229  31.832  -0.071   0.694   0.003   0.303
E E  4EKCA                323    0.591 103.230   0.661   0.782   0.003   0.216
B N  4EKCA                324    0.113  16.514   0.280   0.782   0.003   0.216
B I  4EKCA                325    0.022   4.107   1.371   0.970   0.001   0.030
E R  4EKCA                326    0.358  82.005   1.784   0.970   0.001   0.030
E F  4EKCA                327    0.338  67.837   1.811   0.988   0.000   0.012
B V  4EKCA                328    0.022   3.443   1.029   0.988   0.000   0.012
B F  4EKCA                329    0.036   7.165   0.523   0.988   0.000   0.012
E A  4EKCA                330    0.478  52.709   1.966   0.988   0.000   0.012
B A  4EKCA                331    0.178  19.594   1.265   0.988   0.000   0.012
B V  4EKCA                332    0.017   2.628   1.367   0.988   0.000   0.012
B K  4EKCA                333    0.176  36.265   1.196   0.988   0.000   0.012
E D  4EKCA                334    0.382  55.118   1.331   0.988   0.000   0.012
B T  4EKCA                335    0.094  13.052   1.216   0.988   0.000   0.012
B I  4EKCA                336    0.026   4.736   0.970   0.988   0.000   0.012
B L  4EKCA                337    0.123  22.503   0.838   0.970   0.001   0.030
E Q  4EKCA                338    0.412  73.547   1.288   0.970   0.001   0.030
E L  4EKCA                339    0.384  70.365   1.376   0.923   0.002   0.076
B N  4EKCA                340    0.117  17.085   0.187   0.923   0.002   0.076
B L  4EKCA                341    0.125  22.979  -0.346   0.858   0.002   0.139
E K  4EKCA                342    0.534 109.844   0.871   0.858   0.002   0.139
E E  4EKCA                343    0.668 116.735   0.672   0.782   0.003   0.216
B Y  4EKCA                344    0.214  45.646   0.000   0.406   0.004   0.590
E N  4EKCA                345    0.602  88.162  -0.809   0.109   0.005   0.886
B L  4EKCA                346    0.192  35.082  -0.197   0.018   0.019   0.964
E V  4EKCA                347    0.577  88.685  -1.451   0.003   0.003   0.994

Explain the output.  Go back.
```

---
